# Supplementary material for: Multi‐dataset identification of innovative feature genes and molecular mechanisms in keratoconus
Source: J Cell Mol Med. 2024 Sep 19;28(18):e70079. doi: 10.1111/jcmm.70079 (PMC11412914; doi:10.1111/jcmm.70079)
Supplement: Supplementary file 2 — Material S2. [file JCMM-28-e70079-s002.zip › 7.GSEA analysis/2.ATOH7/KEGG_INOSITOL_PHOSPHATE_METABOLISM.html]

Details for gene set KEGG\_INOSITOL\_PHOSPHATE\_METABOLISM[GSEA]

|  || Dataset | ATOH7.group.cls#h\_versus\_l |
| Phenotype | group.cls#h\_versus\_l |
| Upregulated in class | h |
| GeneSet | KEGG\_INOSITOL\_PHOSPHATE\_METABOLISM |
| Enrichment Score (ES) | 0.40512168 |
| Normalized Enrichment Score (NES) | 1.5656538 |
| Nominal p-value | 0.04291045 |
| FDR q-value | 0.2763907 |
| FWER p-Value | 0.676 |
Table: GSEA Results Summary

  

Fig 1: Enrichment plot: KEGG\_INOSITOL\_PHOSPHATE\_METABOLISM      
 Profile of the Running ES Score & Positions of GeneSet Members on the Rank Ordered List

  

| SYMBOL | TITLE | RANK IN GENE LIST | RANK METRIC SCORE | RUNNING ES | CORE ENRICHMENT || 1 | PIP5K1B | na | 2 | 1.293 | 0.1970 | Yes |
| 2 | PLCE1 | na | 730 | 0.493 | 0.2512 | Yes |
| 3 | PLCB1 | na | 1718 | 0.367 | 0.2787 | Yes |
| 4 | ISYNA1 | na | 2177 | 0.331 | 0.3159 | Yes |
| 5 | PIP5K1A | na | 3253 | 0.267 | 0.3256 | Yes |
| 6 | ITPKB | na | 5756 | 0.178 | 0.2806 | Yes |
| 7 | ITPKA | na | 5860 | 0.175 | 0.3043 | Yes |
| 8 | ITPK1 | na | 6046 | 0.170 | 0.3248 | Yes |
| 9 | INPP5B | na | 6107 | 0.168 | 0.3487 | Yes |
| 10 | IMPA2 | na | 6534 | 0.156 | 0.3602 | Yes |
| 11 | PLCD4 | na | 7237 | 0.137 | 0.3609 | Yes |
| 12 | SYNJ2 | na | 8457 | 0.109 | 0.3423 | Yes |
| 13 | MIOX | na | 8558 | 0.107 | 0.3558 | Yes |
| 14 | PIK3C2G | na | 9044 | 0.098 | 0.3567 | Yes |
| 15 | INPP4A | na | 9302 | 0.094 | 0.3636 | Yes |
| 16 | OCRL | na | 9751 | 0.085 | 0.3637 | Yes |
| 17 | PIP4K2A | na | 9756 | 0.085 | 0.3766 | Yes |
| 18 | PI4KB | na | 9772 | 0.085 | 0.3891 | Yes |
| 19 | PIP4K2C | na | 10060 | 0.080 | 0.3931 | Yes |
| 20 | PI4KA | na | 10067 | 0.080 | 0.4051 | Yes |
| 21 | CDIPT | na | 11392 | 0.061 | 0.3762 | No |
| 22 | PLCZ1 | na | 12549 | 0.045 | 0.3497 | No |
| 23 | PIK3C3 | na | 13157 | 0.039 | 0.3381 | No |
| 24 | TPI1 | na | 14080 | 0.030 | 0.3161 | No |
| 25 | PIP5K1C | na | 14534 | 0.025 | 0.3069 | No |
| 26 | ALDH6A1 | na | 14545 | 0.025 | 0.3105 | No |
| 27 | INPP5A | na | 14761 | 0.023 | 0.3078 | No |
| 28 | IMPA1 | na | 15468 | 0.017 | 0.2900 | No |
| 29 | PIK3CD | na | 16202 | 0.011 | 0.2706 | No |
| 30 | PIK3C2A | na | 16501 | 0.009 | 0.2633 | No |
| 31 | INPP5E | na | 16680 | 0.007 | 0.2593 | No |
| 32 | INPPL1 | na | 17653 | -0.001 | 0.2314 | No |
| 33 | PIK3C2B | na | 17796 | -0.002 | 0.2277 | No |
| 34 | INPP5K | na | 18962 | -0.013 | 0.1960 | No |
| 35 | PLCD1 | na | 19875 | -0.021 | 0.1728 | No |
| 36 | PIK3CB | na | 20685 | -0.028 | 0.1539 | No |
| 37 | PLCB4 | na | 20753 | -0.029 | 0.1564 | No |
| 38 | PIK3CA | na | 21359 | -0.036 | 0.1443 | No |
| 39 | PLCD3 | na | 22176 | -0.045 | 0.1276 | No |
| 40 | PIKFYVE | na | 22283 | -0.046 | 0.1316 | No |
| 41 | PTEN | na | 23399 | -0.060 | 0.1087 | No |
| 42 | PIP4K2B | na | 23746 | -0.066 | 0.1087 | No |
| 43 | INPP5J | na | 23807 | -0.067 | 0.1172 | No |
| 44 | IPPK | na | 24033 | -0.070 | 0.1214 | No |
| 45 | PLCB3 | na | 24121 | -0.071 | 0.1298 | No |
| 46 | IPMK | na | 24144 | -0.072 | 0.1401 | No |
| 47 | PLCG2 | na | 24577 | -0.078 | 0.1395 | No |
| 48 | PLCG1 | na | 25238 | -0.090 | 0.1341 | No |
| 49 | MINPP1 | na | 25597 | -0.096 | 0.1385 | No |
| 50 | PIK3CG | na | 26210 | -0.109 | 0.1374 | No |
| 51 | INPP1 | na | 26326 | -0.111 | 0.1511 | No |
| 52 | PLCB2 | na | 28336 | -0.160 | 0.1176 | No |
| 53 | SYNJ1 | na | 28821 | -0.174 | 0.1301 | No |
| 54 | INPP4B | na | 31407 | -0.264 | 0.0958 | No |
Table: GSEA details [plain text format]

  

Fig 2: KEGG\_INOSITOL\_PHOSPHATE\_METABOLISM      
 Blue-Pink O' Gram in the Space of the Analyzed GeneSet

  

Fig 3: KEGG\_INOSITOL\_PHOSPHATE\_METABOLISM: Random ES distribution      
 Gene set null distribution of ES for **KEGG\_INOSITOL\_PHOSPHATE\_METABOLISM**
